# Rapid phenotyping towards personalized malaria medicine

**DOI:** 10.1186/s12936-020-3149-4

**Published:** 2020-02-11

**Authors:** Maria Isabel Veiga, Weng Kung Peng

**Affiliations:** 1 Life and Health Sciences Research Institute (ICVS), School of Medicine, University of Minho, Braga, Portugal; 2 ICVS/3B’s-PT Government Associate Laboratory, Braga/Guimarães, Portugal; 3 Precision Medicine–Engineering Group, International Iberian Nanotechnology Laboratory, Braga, Portugal

**Keywords:** Malaria control and elimination, Technological innovations, Personalized medicine

## Abstract

Malaria is major public health concerns which continues to claim the lives of more than 435,000 people each year. The challenges with anti-malarial drug resistance and detection of low parasitaemia forms an immediate barrier to achieve the fast-approaching United Nations Sustainable Development Goals of ending malaria epidemics by 2030. In this Opinion article, focusing on the recent published technologies, in particularly the nuclear magnetic resonance (NMR)-based diagnostic technologies, the authors offer their perspectives and highlight ways to bring these point-of-care technologies towards personalized medicine. To this end, they advocate an open sourcing initiative to rapidly close the gap between technological innovations and field implementation.

## Background

Malaria continues to claim the lives of more than 435,000 people each year. The challenges with anti-malarial drug resistance and detection of low parasitaemia form immediate barriers to achieve the fast-approaching United Nations Sustainable Development Goals of ending malaria epidemics by 2030 [1].

Critical for the control and elimination of malaria is an accurate diagnosis of the disease. Malaria infection often goes asymptomatic due to undetectable low parasitaemia is in fact inevitably underestimating the number of malaria carriers. Detecting and treating these infections are of utmost importance to ensure patient health and to block disease transmission, since they are a reservoir for future infections. Light microscopy (LM) remains the standard of practice for malaria diagnosis in febrile patients at health facilities, nevertheless this method requires well-trained personnel and does not adequately detect low parasitaemia (detection limit: ~ 50 parasite/μL of blood) [2]. Alternatively, rapid diagnostic test (RDTs), based on immunochromatographic test are ready-to-use assays and have facilitated access to malaria diagnosis even at the community level. Its simplicity to perform and interpret, allowed a significant increase of the proportion of patients suspected of having malaria in receiving a malaria diagnostic test (59% from 2015– 2017, up from a median of 33% for the period 2010–2012) [1].

Nonetheless, RDTs are not more sensitive than LM and, similarly, cannot detect the low-level blood-stage malaria infections that can otherwise be identified by genetic methods [3]. Nucleic acid amplification-based tests (NAATs), such as polymerase chain reaction (PCR) and loop mediated isothermal amplification (LAMP) have been emerging as conceivable option with much greater sensitivity (down to 1 parasite/µL of blood) in diagnosis [4]. Nevertheless, it still requires minimal laboratory conditions, advanced staff training, relatively long time-to-results (30–60 min) and high costs, limiting its usefulness in resource-limited settings. In response to this needs, highly sensitive point-of-care tests such as ultra-sensitive RDTs [5] (already commercially available), paper-based microfluidics for deoxyribonucleic acid (DNA) detection [6] or even nuclear magnetic resonance (NMR)-based hemozoin detection [7–10], has been developed for malaria diagnostics. The use of this highly sensitive point-of-care tests are crucial to support malaria elimination and have been considered and discussed under the Malaria Policy Advisory Committee [11]. In this Opinion article, we focus on the NMR-based hemozoin detection technologies as diagnostic test that not only can deliver sensitivity but also its potentiality towards malaria personalized malaria medicine.

### NMR-based hemozoin detection

A rapid malarial screening has been recently reported based on point-of-care NMR system [7, 9, 10, 12]. NMR spectroscopy is a well-established, non-invasive technique for biochemical, structural studies and a powerful approach in characterizing metabolic responses in biological samples. NMR-based malaria detection is centered on recognizing the paramagnetic susceptibility of malaria hemozoin crystals, which conditions a differential proton nuclear magnetic resonance signature of the infected erythrocyte. This in turn allows a quantitative determination of the parasitaemia. Being parasite survival dependent on hemozoin formation as a by-product of haem detoxification process upon haemoglobin degradation, makes this natural marker, a good unique feature to identify parasites presence in patients’ blood.

As shown in mice studies, the micro NMR system has ‘hypersensitivity’ with a detection limit of less than 10 parasite/µL of blood [7]. This could be a game-changer in resource limited-settings since advance in technology made it portable, needs only one drop of blood (same as microscopy or RDT sample test), and results in less than 10 min. The research team further demonstrated that low-cost, and much stable detection baseline was possible by concentrating the less deformable infected red blood cells using microfluidic separation technique. This high level of sensitivity challenges its usefulness to single out asymptomatic patients or to conduct large randomized screening in the field. In malaria pre-elimination setting, detection of asymptomatic parasite carriers is detrimental since this often low parasite density infections, constitute a large proportion of the parasite reservoirs. Clearing this parasite reservoir is critical for malaria elimination as these individuals still remain infectious to the mosquito promoting the disease transmission. Therefore, NMR-based technology revealed with high potential to be used as high-throughput sensitive diagnostic tool capable to be used in field limited settings.

### Sensitivity versus specificity

While good NMR sensitivity (‘true positive rate’) has been reported, the issue of specificity (‘false positive rate’) remains elusive [7, 8]. Hemozoin is also the signature of other blood-borne parasites (e.g., *Schistosoma*), favoring their difference in disease manifestation symptoms to distinguish them. One far more relevant problem would be to distinguish *Plasmodium* species once treatment is usually administered based on the *Plasmodium* species detected. There are five *Plasmodium* species that can infect humans, being *Plasmodium falciparum* responsible for the highest mortality rate [13] and *Plasmodium vivax* responsible for more than 2.5 billion people at risk of infection [1, 14]. The process of haemoglobin degradation, in all *Plasmodium* species, right from the starting of parasites invasion in the intraerythrocytic cycle leads to hemozoin crystals formation, but their shape and condensation differs [15].

The process of hemozoin formation is mediated heavily by the transfer of electron. Sienkiewicz had shown using multi-frequency high field electron paramagnetic resonance (EPR) that the spectra obtained for hemozoin and synthetic β-haematin can only correspond to a high spin Fe-III (S = 5/2) [16]. The presence of unpaired electrons can be used as EPR-marker to unveil possibly much more intermediate steps, in this poorly understood arena. There is a window of opportunity in which one can manipulate the electron spin resonance using EPR along with the current NMR detection [17] or any of the electron-nuclei double resonance (ENDOR) variants [18]. The small but significant hyperfine interactions from the electron-nuclear coupling allows enormous information to be mapped out. Thus, the use of EPR, ENDOR or multidimensional NMR spectroscopy should have the capacity to differentiate the detailed structure of hemozoin [19] leading to species identification.

### Towards the NMR-based personalized malaria medicine

Taking NMR technology beyond parasite detection, this technology has high potential to address another important factor that hampers the control and elimination of malaria i.e., the rapid parasite drug resistance acquisition. Scaling up to higher dimension (e.g., two- or multi-dimensional), NMR spectroscopy may provide the capability of detecting parasite ‘phenotypic variation’. Multi-dimensional representation would essentially means obtaining multifactorial markers (a panel of associated biomarkers) in a single snapshot which in return increases the accuracy of diagnostic [20, 21]. Obtaining time- and patient-unique ‘*molecular fingerprint*’ is the first step towards personalized malaria medicine, which has vast implication to the malaria elimination programme due the increasingly rate of phenotypic variations in the field [21].

In good time, multi-dimensional NMR techniques are well developed within the NMR community. With the development of fast and highly efficient multidimensional Inverse Laplace decomposition algorithm [22–24], and the recent demonstration of two-dimensional low-field NMR relaxometry applications [24–26] maybe the answer. By decomposing the multiple proton relaxation reservoirs arising from the water-protein interactions in red blood cells (RBCs), one could hypothetically capture the unique phenotypic expression (e.g., physiological patterns) at every stage of infection. Considering valid this hypothesis, there are a number of significant implications, and they are as follow;

Anti-malarial drug resistance has been correlated with specific genetic mutations in the parasite, offering a tool for the development of diagnostic tests to monitor resistance. But the analysis of the genetic maker, usually through nucleic acid amplification and detection systems, demands technical handling expertise and generally above 1-h turnaround time, precluding, up to today, the development of a device/tool that could provide this information at the point-of-care settings. On top of this, there are anti-malarial drugs such as the artemisinin, for which mechanism of action and resistance is yet to be fully disclosed owning to the possible epigenetic involved. As an example, mutations at the *P. falciparum k13* gene does not explain some cases of phenotypic variations, such as parasite clearance time upon artemisinin-based combination therapy (ACT) treatment within the same gene [27, 28]. These phenotypic variations could be based on geographical location or differential transmission setting or even the different ring developmental stage or other genetic factors that needs to work together to reveal a phenotype [29]. Therefore, the NMR or other spectroscopic technologies [25, 30, 31] could aid with the challenges we are facing in detecting anti-malarial drug resistance providing more meaningful information as rapid phenotyping rather than genotyping.

As an outcome of the above mentioned, the anti-malarial drug resistance could hypothetically be captured by multidimensional spectroscopy without a priori knowledge (hypothesis free) on the genetic markers or even independent of other -omics details. Anti-malarial target different features of the parasite’s biology leading to changes in metabolic responses. We hypothesized that the alteration of protein chemistry in the infected RBCs with different anti-malarial susceptible parasites could lead to a distinctive spectrum due to the sensitivity of water-protein interactions in the time-domain multidimensional NMR.

### Future outlook: the silver lining

Parasites were first detected under the microscope in 1884 by Alphonse Laveran, a military doctor in France’s Health Service of the Armed Forces. It has passed now almost a century and a half since its discovery and imposingly, microscopy continues to be the recommended method for malaria diagnosis in the field.

Spectroscopic techniques (e.g. NMR, surface enhanced Raman spectroscopy (SERS)), and ultralow cost microfluidic chip [6, 10, 32–35] are starting to gain the momentum in this joint multidisciplinary expertise to deliver much more sensitive and rapid field malaria detection. We herein disclose the potentiality of going beyond detection and detail some NMR characteristics that could take us towards personalized malaria medicine in low resources settings. In fact, these technologies visioning point of care devices are also moving towards the tantalizing idea of non-invasive, needle free detection. Characteristics such as reliably diagnose with no skin puncture and consequently with no disposable wastes, meets the growing field use medical and environment demands [36]. Conceptualizing the NMR as point of care device, a low-cost wearable device (e.g., pulse oximetry) or compact body fluids analyser for monitoring may become feasible in near future but remain a foreseen test. This may be possible if engineers can step up the game by using the radio/microwave or infrared spectrum as transducer, in analogous to the existing (and already viable) technologies of micro magnetic resonance imaging [37] or SERS [38], respectively.

Far too often, we found ideas and proposal being thrown off beyond publications in this tight funding environment, and unwillingness of investors to put in their money in what often dubbed as the ‘*poor*-*man disease*’. In order to accelerate this cause, we need researchers to open-source code (e.g., hard/software) and open-source the ideas/solutions, so that it will continue to spark innovation in the place where the technologies are needed most. With ubiquitous access to internet as meeting place (e.g., open access articles), and low-cost fabrication of hardware (e.g., Arduino, 3D printing), more practical solution can be brought to doorstep. The OPENCORE NMR project [39] by K. Takeda for example, has lowered the barrier of building NMR spectrometer tremendously. The emergence of software-defined-radio (SDR) [21, 40, 41] is making what once used to be expensive radio pulser/receiver within the reach of hobbyist now. SDR enables the replacement of traditional hardware of radio-frequency components with software-based signal processing. Such a movement has the potential to shift the research community and society at large towards technology democratization, departing from the (over)-dependency on expensive commercial spectrometers.

There is a silver lining in this: having low-cost instrumentation with ultra-low-cost assays for malaria diagnostic (and monitoring), will then immediately translate the know-how (e.g., spectroscopic techniques, or discovery of new markers) for other infectious diseases or even other chronic diseases aka the ‘rich-man diseases’ (e.g., diabetes mellitus [31], and cancer [42]) in developed countries.

## Conclusion

Accurate diagnosis of malaria and the resilient capacity that the malaria parasite has in acquiring resistance to anti-malarial drugs form immediate barriers to the control and elimination of this disease. Researchers in the field are beginning to see more cases of phenotypic variations. These phenotypic variations could be based on geographical location or differential transmission setting or even the different ring developmental stage [28, 30]. In these regards, we discussed the recent advances in spectroscopic-based technologies which is able to reveal unique ‘molecular fingerprint’, and thus providing the much needed rapid phenotyping (rather than genotyping) platform in the field [20, 40].

## Data Availability

Data sharing not applicable to this article as no datasets were generated or analysed during the current study.

## Abbreviations

NMR: Nuclear magnetic resonance
LM: Light microscopy
RDTs: Rapid diagnostic test
NAATs: Nucleic acid amplification-based tests
PCR: Polymerase chain reaction
LAMP: Loop mediated isothermal amplification
DNA: Deoxyribonucleic acid
EPR: Electron paramagnetic resonance
ENDOR: Electron-nuclei double resonance
RBCs: Red blood cells
ACT: Artemisinin-based combination therapy
SERS: Surface enhanced Raman spectroscopy
SDR: Software-defined-radio
